# Podoconiosis: key priorities for research and implementation

**DOI:** 10.1093/trstmh/traa094

**Published:** 2020-11-09

**Authors:** Kebede Deribe, Charles D Mackenzie, Melanie J Newport, Daniel Argaw, David H Molyneux, Gail Davey

**Affiliations:** Brighton and Sussex Centre for Global Health Research, Department of Global Health and Infection, Brighton and Sussex Medical School, Brighton BN1 9PX, UK; School of Public Health, College of Health Sciences, Addis Ababa University, PO Box 9086, Addis Ababa, Ethiopia; Taskforce for Global Health, Decatur, GA 30030, USA; Brighton and Sussex Centre for Global Health Research, Department of Global Health and Infection, Brighton and Sussex Medical School, Brighton BN1 9PX, UK; World Health Organization, Control of Neglected Tropical Diseases, Geneva 1211, Switzerland; Liverpool School of Tropical Medicine, Liverpool L3 5QA, UK; Brighton and Sussex Centre for Global Health Research, Department of Global Health and Infection, Brighton and Sussex Medical School, Brighton BN1 9PX, UK; School of Public Health, College of Health Sciences, Addis Ababa University, PO Box 9086, Addis Ababa, Ethiopia

**Keywords:** diagnosis, lymphoedema, podoconiosis, research priorities, treatment

## Abstract

Podoconiosis is a non-infectious tropical lymphoedema causing swelling of the lower legs. Podoconiosis is associated with stigma, depression and reduced productivity, resulting in significant socio-economic impacts for affected individuals, families and communities. It is caused by barefoot exposure to soils and affects disadvantaged populations. Evidence from the past 5 y suggests that podoconiosis is amenable to public health interventions, e.g. footwear and hygiene-based morbidity management, which reduce acute clinical episodes. Although much has been learned in recent years, advances in care for these patients and worldwide control requires further reliable and relevant research. To develop a comprehensive global control strategy, the following key research priorities are important: better understanding of the global burden of podoconiosis through extended worldwide mapping, development of new point-of-care diagnostic methods and approaches to define the presence of the environmental characteristics that contribute to the development of the condition, improving treatment through an increased understanding of the pathogenesis of dermal changes over time, improved understanding of optimal ways of providing patient care at the national level, including research to optimize behavioural change strategies, determine the optimum package of care and integrate approaches to deliver robust surveillance, monitoring and evaluation of control programmes.

## Introduction

Podoconiosis is a disabling form of lymphoedema that, through recurrent painful episodes of acute dermatolymphangioadenitis and physical deformity, reduces productive working days among subsistence farmers by 45% each year,^1,2^ causing progressive lymphoedema of the lower limb. Affected people are highly stigmatized,^3,4^ experience depression^5^ and face barriers to education, employment and a range of social interactions, including marriage.^3^ In 2011 the World Health Organization identified podoconiosis as one of the neglected tropical diseases (NTDs).^6^ The disease, which affects genetically susceptible individuals who go barefoot, is linked to long-term exposure to red clay soil.^7^ Interactions between genetic and environmental factors trigger an inflammatory response that leads to lymphoedema and fibrosis.^8^ Here we review recent progress in understanding this important clinical condition and identify the operational research questions (Box 1) that will support the further development of global control and elimination strategies.

Box 1. Key research questions

Basic sciences

- Which mineral particles in the soil are responsible for causing podoconiosis?
- What immunological changes occur as a result of podoconiosis?
- What pathological changes occur in the human lymphatic system due to podoconiosis?

Diagnosis

- What is the accuracy and reproducibility of diagnosis of podoconiosis using a clinical algorithm in different epidemiological settings?
- Can spectroscopy combined with machine learning be used for the diagnosis of podoconiosis or to predict the risk of podoconiosis in the preclinical stage?
- What molecular test might be used to diagnose podoconiosis?

Pathogenesis

- Are there specific changes in the vascular and dermal tissues that are specific and relate to the development of new therapeutic approaches?
- What is the role of the dermal–epidermal disruption in the pathogenesis and progression of podoconiosis?

Epidemiology and mapping

- What is the population at risk and number of cases in the 14 countries with documented evidence of podoconiosis?
- What is the current status of the 15 historically endemic countries?
- How can podoconiosis surveys be integrated with other NTDs and health surveys?
- How can podoconiosis be best included in routine national reporting systems?

Disease burden

- What is the global burden of podoconiosis?
- What is the economic cost of control and elimination of podoconiosis?

Treatment

- What is the community-level impact of hygiene-based morbidity management?
- How might adenolymphangitis be better characterized and diagnosed?
- Does the addition of doxycycline to hygiene-based management arrest progression of the disease or reduce the incidence of adenolymphangitis?
- What other interventions applied to the management of lymphoedema in high-income counties could be added to hygiene-based management?
- What is the optimal duration of supervised hygiene-based morbidity management?

Prevention and control strategies

- Which behavioural interventions are most effective in promoting sustained changes in preventive behaviour?
- Under what circumstances should community-based rather than facility-based morbidity management be provided?
- What is the optimal package of care for people with podoconiosis?
- What is the optimal surveillance, monitoring and evaluation strategy for interventions against podoconiosis?
- How might podoconiosis control be integrated with that of other NTDs and with other health programmes?

## Disease burden, pathogenesis, epidemiology and mapping

Podoconiosis is caused by exposure to mineral particle–induced inflammation on a background of genetic susceptibility.^1^ Interactions between genetic and environmental factors trigger an inflammatory response that leads to lymphoedema and fibrosis.^1^ It is hypothesized that mineral particles that penetrate bare skin are engulfed by macrophages in the lower limb lymphatics and induce an inflammatory response in the lymphatic vessels. This is followed by fibrosis and obstruction of the vessel lumen, leading to oedema of the lower leg, which may progress to lymphoedema.^9^ A much better understanding of the pathogenesis of podoconiosis, focussing on changes in the skin and the lymph system, are critical in order to develop diagnostics and better treatment. For example, are the pathological changes in the lymphatic vessels and system similar to those proposed in lymphoedema of parasitic origin (lymphatic filariasis [LF])? In the latter condition, it is thought that the parasite induces hyperproliferation of the lymphatic vessels, leading to vascular incompetence. Knowledge of temporal changes to the anatomy and drainage efficiency of the lymph vessels should allow for improved therapeutic measures and diagnostic interpretation of early changes. The nature of functional changes in the dermal–epidermal tissues in the affected region could also be informative.^10,11^ With the more extensively studied lymphoedema in filariasis it is now thought that the maintenance of upper dermis and epidermis is crucial to healing and recovery.^10,11^ The skin is known to be a major source of active protective and sometimes inflammatory molecules, for example, in its role as a component of the body's innate immunity. Understanding changes in the skin as podoconiosis develops and following treatment are fertile areas for investigation and could in all likelihood contribute to developing and monitoring new therapeutic approaches. Studies that will elucidate further the genetic basis of susceptibility and the pathogenic pathways the minerals trigger should be continued and extended.

**Figure 1.**
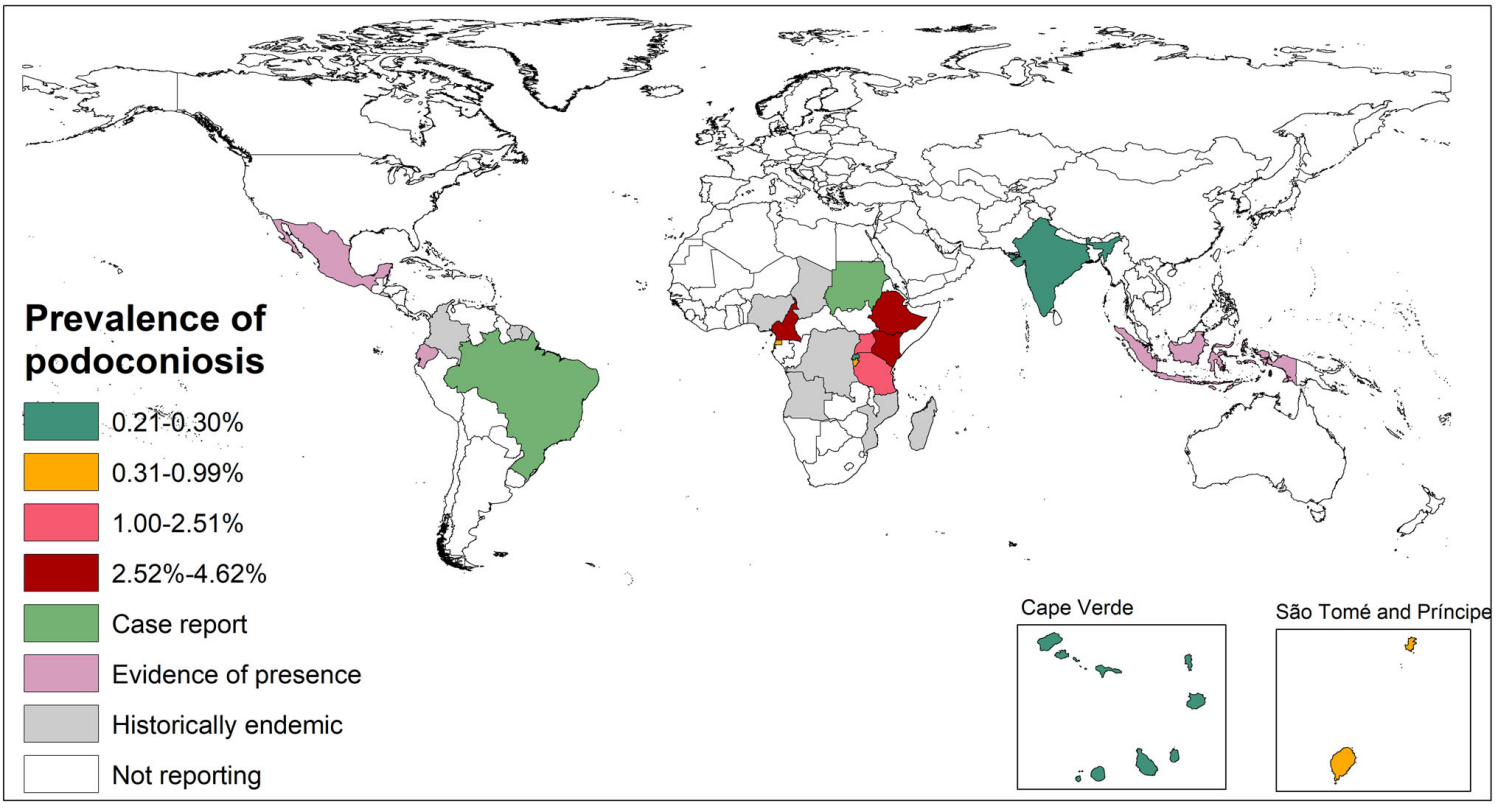
Global distribution and prevalence of podoconiosis. This figure was adapted from Deribe et al.^13^ and is available for distribution under the terms of the Creative Commons Attribution Licence, which permits unrestricted use, distribution and reproduction in any medium.

Globally it is estimated that there are about 4 million people with podoconiosis, mainly in tropical Africa, Central and South America and Southeast Asia.^12,13^ Tropical African countries bear the highest disease burden.^14–16^ A recent systematic review identified 32 countries where the disease has been reported either currently or historically.^13^ Another study, which used a combination of evidence sources, concluded that there is evidence of podoconiosis in 17 countries (12 in Africa, 3 in Latin America and 2 in Asia) and consensus on its presence in a further 6 countries (all in Africa) (Figure 1).^17^ So far only three countries (Cameroon, Ethiopia and Rwanda) have conducted nationwide mapping of podoconiosis as part of developing the global atlas of the disease.^12,16,18,19^ A scalable mapping approach utilizing mobile phone and geographic information system technologies has been developed and applied in these three countries.^16,18,20^ In addition to community-based surveys, the mapping approach uses remote sensing advances to produce datasets that define areas at risk of podoconiosis using a combination of climate, environmental and ecological parameters.^21^ The approach enables clear definition of the geographical distribution of the disease and estimation of the population at risk and the number of individuals affected, which helps prioritize implementation efforts.^14–16,21^ It is critical that mapping is completed in the remaining countries in which there is clear evidence of podoconiosis.

Estimating the global burden of podoconiosis is vital. Such data provide policy makers, programme planners and clinical practitioners clear burden estimates that help in priority setting and decision making. Strong country-level data spur engagement of endemic countries’ medical and public health communities and prompt national implementation. A study conducted in Ethiopia estimated the disability-adjusted life years (DALYs) due to podoconiosis sequelae (lymphoedema and acute attack) to be 172 073, higher than the DALYs due to other NTDs, including trachoma, onchocerciasis and leishmaniasis.^22^ Studies have clearly shown an association between depression and podoconiosis, one documenting a prevalence of depression of 12.6% among people with podoconiosis compared with 0.7% among healthy neighbours.^5^ In the future, burden estimation should establish podoconiosis-appropriate disability weights and include anxiety and depression as a further sequela of podoconiosis (Box 1).

Some of the environmental factors that facilitate formation of the red clay soils containing the putative inorganic particles that trigger the inflammatory response leading to podoconiosis have been described.^23^ The exact causal agent in red clay soil areas has not yet been identified. A range of studies has suggested that mineral particles present in red clay soils play a role in the pathogenesis of podoconiosis.^24–28^ Electron microscopy microanalysis of lower limb lymphatic tissues of barefoot people from Ethiopia indicated the presence of microparticles containing the elements found in clays, with some difference in the amount between affected and unaffected individuals.^29^ Studies to better characterize the types of soils and soil particles in diverse geographical areas are required.

In the early 1970s, Price^9^ reported familial aggregation of podoconiosis in Ethiopia, Rwanda and Burundi. These observations and subsequent epidemiological studies in Ethiopia hinted at a high heritability of podoconiosis.^30–33^ Price performed segregation analysis, which suggested the possibility of a genetic factor with an autosomal recessive mode of inheritance.^34^ Subsequently it was hypothesized that individual differences in the tissue handling of absorbed minerals play a role in the development of full-blown podoconiosis.^35^ A pedigree study conducted in 2005 in southern Ethiopia illustrated that both genetic and environmental factors contribute to the pathophysiology of podoconiosis.^8^ A genome-wide comparison of the frequency of genetic variants between podoconiosis cases and unaffected controls from southern Ethiopia revealed that genetic variants in the human leucocyte antigen locus (a genomic region on chromosome 6) confer susceptibility to podoconiosis. The study suggested that podoconiosis is a T cell–mediated inflammatory condition.^7^ Further studies to replicate these findings in different populations are required.

## Diagnosis

Currently podoconiosis diagnosis is primarily clinical and based on history, physical examination and certain disease-specific tests to exclude common differential diagnoses.^20^ Current ongoing studies include developing a clinical algorithm, testing and adapting a portable, three-dimensional, infrared imaging system^36,37^ and using bioelectrical impedance analysis^38^ to detect early, and therefore reversible, lymphoedema due to podoconiosis. The clinical algorithm depends on history and physical examination, suggesting that detailed phenotyping might be an important preliminary step. Although it has high sensitivity and specificity, validation in a range of epidemiological and geographical settings will be required and should be accompanied by the development of training materials. The use of geographical characteristics such as altitudinal difference with LF is important given the altitudinal limits of *Anopheles* vectors of filariae (and malaria) transmission around 1700 m.^20^

Innovation in the diagnosis of podoconiosis is important to accelerate the mapping, scale-up of interventions and ultimately elimination of podoconiosis. Spectroscopy combined with machine learning has been used to predict the risk of diseases,^39^ to accurately distinguish between vertebrate blood meals in the guts of malaria mosquitoes^40,41^ and to identify sources of dog diets.^42^ The method has also been applied for studying the vibrational fingerprint of organic compounds through imaging analysis. This approach could, in the future, be applied to podoconiosis, possibly through scanning the skin and lymphatic system changes or using non-invasive procedures such as hair samples. The application of such technology could have important implications for the diagnosis of podoconiosis in the short term. Nonetheless, the long-term goal should be a point-of-care diagnostic for podoconiosis.

A point-of-care diagnostic tool is critical for rapid scale-up of mapping and interventions in endemic countries. Identifying potential biomarkers is important to achieve this goal. We hypothesize that as the pathogenesis of podoconiosis is likely to involve activation of monocyte–macrophage cells lines (catalysed by mineral elements), determining increases in circulating biomarkers of macrophage activation will be important.^43^

The diagnosis of sequelae is also important. At present, episodes of acute dermatolymphangioadenitis are diagnosed clinically. Standardising clinical definitions and repositioning other approaches such as thermography to detect secondary inflammation will enable improved identification of these episodes.

## Prevention and control strategies

The key strategy for podoconiosis control is prevention of contact with irritant soil through footwear use, foot hygiene and covering floors.^44^ Although important formative work has uncovered many of the barriers to adoption of preventive behaviours,^45,46^ evidence around the effectiveness of behavioural interventions is less conclusive.^47^ Evaluation of behavioural interventions, including community conversations and patient-led groups, will be critical to the development of robust prevention strategies. Continuing to engage social and behavioural scientists in developing robust strategies is critical.

The basic package of care for patients includes treatment for and management of episodes of lymphoedema morbidity, as recommended by World Health Organization (WHO). This significantly improves quality of life by reducing the frequency of acute attacks and preventing disease progression.^48,49^ A recent randomized controlled trial demonstrated that hygiene-based management reduced the incidence of episodes of acute dermatolymphangioadenitis by 20% at the 12-month follow-up.^48^ While the results of the trial are encouraging, the long-term effects and degree of adherence should be studied, as should the community-level impact of the intervention. Efforts to reduce the water requirements of standard morbidity management through the addition of glycerine during foot soaking are promising and should be included in future operational research where glycerine is available.^50^ Morbidity management is one aspect of treatment for people with podoconiosis. There is also a need to test a comprehensive care package addressing the psychosocial and mental health needs of affected individuals in addition to their physical care.

Cross-sectional studies have suggested that doxycycline has beneficial effects among people with LF.^51,52^ A small prospective study in Ghana compared patients with LF treated with either doxycycline, amoxicillin or placebo and followed the progress of their lymphoedema over a 24-month period after treatment.^53^ The study demonstrated that doxycycline arrested progression of the disease and reduced acute episodes. Currently a large-scale study is under way in six countries to investigate the impact of 6 weeks of treatment with doxycycline added to standard limb hygiene on early stage filarial lymphoedema and podoconiosis.^54^ Results will be available in 2021. The rich parallels between skin changes in podoconiosis and LF suggest future opportunities for research into therapeutics that span the interface between these conditions.

Linking podoconiosis research to mainstream clinical dermatology research will be of significant benefit. Innovations being developed for skin conditions with similar dermal–epidermal disruption and fibrotic manifestations may have important impacts on restoring the cutaneous barrier in patients with podoconiosis. This goal has been greatly enhanced by two major issues: first, the considerable amount of fieldwork carried out in the past few years, and second, the new dermatology-focused initiatives of the WHO related to NTDs.^55^ The increased awareness of podoconiosis and the move to integrate issues such as diagnosis and implementation of care for dermatosis seen in the tropical regions of the world describe a situation that is favourable to increased research efforts in all areas of this condition. It is important that dermatologists and researchers seize this opportunity to discover more about podoconiosis and its treatment. One current example of integrating podoconiosis with research into other dermatological NTDs is the ongoing clinical trial to investigate the impact of 6 weeks of treatment with doxycycline added to standard limb hygiene on early stage lymphoedema in six sites in Africa and the Indian subcontinent.^54^

## Programme implementation

The scale of the burden of podoconiosis in endemic countries is so significant that morbidity management must be approached systematically^14,15^ and in an integrated manner. The WHO NTD Roadmap 2021–2030 signals movement away from single disease silos towards integrated programmes.^55^ Currently morbidity management services are mostly provided on a small scale by non-governmental organizations in most endemic countries. Integration of these services into the health system is long overdue and will require certain key programmatic adaptations. It is critical that ministries of health in endemic countries take the lead in designing interventions and implementations. The Ethiopian experience can be taken as an example.^56^ At the national level, podoconiosis was included in the NTDs master plan.^57^ In the national NTDs unit, a focal person responsible for the implementation of podoconiosis was assigned and an integrated morbidity management manual for LF and podoconiosis was developed. Podoconiosis prevention and morbidity management were included in the annual refresher training package for healthcare providers. An indicator to track the number of people treated for lymphoedema was included in the national Health Management Information System.^56^ Podoconiosis interventions were included in the national Essential Health Services Package.^58^ All these are important prerequisites for the integration of morbidity management into the national health system.

In endemic countries, podoconiosis is often misdiagnosed and there are often misconceptions about the causes, prevention and treatment of podoconiosis.^59^ Therefore formative research to understand these misconceptions and service barriers is important to develop tailored messages and behavioural change and social mobilization strategies. Integration of clear, compelling information on podoconiosis into medical, nursing and paramedical curricula will be vital in equipping the next generation of healthcare professionals.

Well-designed implementation research is required to understand how best to integrate podoconiosis interventions into the health system. Implementation research assessing the integration and scale-up of a holistic package of care—including physical health, mental health and psychosocial care—into routine health services for patients with podoconiosis, LF and leprosy is ongoing in northern Ethiopia. The study involves the development of a comprehensive holistic care package, a pilot study conducted in one subdistrict and scale-up of the care package and evaluation in regard to coverage, implementation and economic outcomes.^60^ Rehabilitation services to address the physical and mental health needs of people with podoconiosis are also important. Designing minimum mental health service packages that can be integrated within the morbidity management package is also a priority.

The development of a framework and tools for surveillance, monitoring and evaluation with key programme-level indicators is important. Implementation research on active surveillance based on environmental, behavioural and climatic risk factors and mandatory passive surveillance is also necessary. From the patient side, behavioural change to encourage early presentation for treatment screening will be important. Given the strong heritability of susceptibility to podoconiosis, a family-based approach to interventions targeting those with a family history may be an important entry point. The use of school-based programmes for health education and to initiate early shoe wearing must be tested. Research linked to the implementation of programmes and challenges that programmes face as they mature requires social science involvement to improve coverage and adherence.

Global-level guidance by the WHO is required in terms of what types of intervention and service-delivery modalities are appropriate, based on endemicity level. In high-burden areas, community-level interventions will be essential. Thus guidance for developing community-level service provision is important. In low-prevalence settings, intensified case management services integrated within primary healthcare are sufficient. Supplies required for morbidity management should be included in the essential drug and medical supply lists of endemic countries to stimulate resource mobilization, procurement and distribution. The global economic costs of intervention should also be estimated to build a strong case for investment.

## Conclusions

Much progress in podoconiosis research and intervention has been made over the past 5 y, particularly through careful evaluation of the effectiveness of a morbidity management intervention appropriate for low-resource settings. Preventive interventions addressing behavioural change and development of a mapping strategy have also been important. This review has identified research gaps that include estimating the global burden of podoconiosis, developing point-of-care diagnostics, creating innovative approaches to enhanced treatment, evaluating large-scale community-level control strategies and the need for integration of podoconiosis into medical education. Finally, strong advocacy is required to advance the control and elimination of podoconiosis globally.

## Data Availability

None.
